# Implementation of Exome Sequencing in Prenatal Diagnostics: Chances and Challenges

**DOI:** 10.3390/diagnostics13050860

**Published:** 2023-02-23

**Authors:** Ewa Janicki, Marjan De Rademaeker, Colombine Meunier, Nele Boeckx, Bettina Blaumeiser, Katrien Janssens

**Affiliations:** 1 Faculty of Pharmaceutical, Biomedical and Veterinary Sciences, University of Antwerp, 2000 Antwerp, Belgium; 2 Center for Medical Genetics, University of Antwerp and University Hospital of Antwerp, 2650 Antwerp, Belgium; 3 Center for Medical Genetics, Institut de Pathologie et de Génétique Gosselies, 6041 Charleroi, Belgium

**Keywords:** prenatal diagnosis, whole exome sequencing, chromosomal microarray, diagnostic yield, congenital anomalies

## Abstract

Whole exome sequencing (WES) has become part of the postnatal diagnostic work-up of both pediatric and adult patients with a range of disorders. In the last years, WES is slowly being implemented in the prenatal setting as well, although some hurdles remain, such as quantity and quality of input material, minimizing turn-around times, and ensuring consistent interpretation and reporting of variants. We present the results of 1 year of prenatal WES in a single genetic center. Twenty-eight fetus-parent trios were analyzed, of which seven (25%) showed a pathogenic or likely pathogenic variant that explained the fetal phenotype. Autosomal recessive (4), de novo (2) and dominantly inherited (1) mutations were detected. Prenatal rapid WES allows for a timely decision-making in the current pregnancy, adequate counseling with the possibility of preimplantation or prenatal genetic testing in future pregnancies and screening of the extended family. With a diagnostic yield in selected cases of 25% and a turn-around time under 4 weeks, rapid WES shows promise for becoming part of pregnancy care in fetuses with ultrasound anomalies in whom chromosomal microarray did not uncover the cause.

## 1. Introduction

Major congenital anomalies (MCA) have a prevalence of 2–3% and are responsible for a significant percentage of pre- and perinatal demise and neonatal morbidity [1,2]. The etiology is heterogeneous, ranging from prenatal infections over teratologic agents to genetic causes.

Chromosomal microarray analysis (CMA) has been widely implemented in the analysis of invasively obtained prenatal samples (amniotic fluid or chorion villi) for the genome-wide detection of both aneuploidies and microdeletions/microduplications (copy number variants or CNVs). In up to 40% of pregnancies with a fetal structural anomaly, CMA is able to diagnose an aneuploidy or CNV [3], still leaving more than half of the cases undiagnosed.

Several recent metaanalyses have demonstrated an added diagnostic yield of 1.8–68% for prenatal whole exome sequencing (WES), with the yield largely depending on the inclusion criteria and organ system affected [4,5,6,7,8]. With increasing evidence of the relevance of WES in the prenatal context, revision of the guidelines of the International Society for Prenatal Diagnosis (ISPD) offers directions on how to implement it [9].

This paper describes the experiences of a single Belgian genetic center with the implementation of WES in the prenatal diagnostic workflow. In Belgium, the molecular analysis of publicly funded invasive prenatal diagnosis can only be executed at one of the eight Centers for Medical Genetics. For all indications, a genome-wide microarray is performed with national consensus guidelines in place steering the interpretation and reporting of the results [10]. Recently, a national framework has been formulated guiding the indication, analysis and reporting of prenatal WES.

Here we discuss the opportunities and challenges for the use of WES in the diagnosis of fetuses with ultrasound abnormalities and provide suggestions for implementation of this valuable technique in other labs.

## 2. Methods

Genomic DNA was extracted from either amniotic fluid, chorion villi or cultured amniocytes using the Maxwell RSC Blood DNA kit on a Maxwell RSC 48 Instrument (Promega, Madison, MI, USA). Library prep on 50ng of genomic DNA was performed using the Twist Human Core Exome kit (Twist Bioscience, South San Francisco, CA, USA) according to the manufacturer’s instructions on a Hamilton STAR robot (Hamilton, Bonaduz, Switzerland). Twenty-four libraries were pooled equimolarly for sequencing on a NextSeq500 or NextSeq550 instrument with a 2 × 75 bp or 2 × 150 bp flow cell (Illumina, San Diego, CA, USA). WES data were analyzed using an in-house developed pipeline which considers only de novo, X-linked and recessive variants, either in a predefined panel (e.g., in case of a skeletal dysplasia) or exome-wide [11]. Additionally, the AI-driven decision-support software Moon was applied to complement our pipeline with an independent phenotype-driven analysis (Invitae, San Francisco, CA, USA), allowing the identification of variants outside the panel (if applied) and of inherited variants. An independent analysis was performed to detect sample swaps and to verify the family relations within each trio.

The guidelines for prenatal WES were developed at a national level and can be found at the website of the Belgian College of Genetics (www.college-genetics.be (accessed on 1 December 2022)). The following criteria must be met: (1) The fetus shows ultrasound anomalies, but CMA is negative; a diagnosis is essential to guide the pregnancy/neonatal management; (2) All cases should be reviewed in a multidisciplinary team including a clinical geneticist; (3) Expert fetal ultrasound examinations are required to provide the best possible phenotypic evaluation. When beneficial, fetal MRI may be performed; (4) Pretest counseling by a clinical geneticist is mandatory, with signed informed consent by both parents; (5) Trio analysis (simultaneous analysis of the fetus and both parents) is strongly recommended to speed up the process. Variant classification is performed based on the ACMG guidelines [12]. Only pathogenic (class V) and likely pathogenic (class IV) variants with known effect on gene function and which fit with the fetal phenotype and the inheritance mode are communicated. Variants of uncertain significance (class III) are in principle not communicated, but exceptions can be made for variants in known disease genes that (a) fit the fetal phenotype, (b) are expected to show the same pathomechanism as known pathogenic variants and (c) arose as de novo events or are detected *in trans* with a pathogenic or likely pathogenic variant and for which further clinical exams (ultrasound, MRI, etc.) are recommended to refine variant classification, possibly leading to a genetic diagnosis (upgrade of the variant to class IV/V). By national agreement, no systematic search for secondary findings, unrelated to the fetal phenotype, is performed, in line with the framework proposed by Vears et al. [13] The identification of incidental findings is minimized by optimizing the filter settings without jeopardizing the detection of primary results. In this category, de novo fetal highly penetrant class IV/V variants known to cause moderate or severe childhood-onset disorders are reported, as well as inherited class IV/V variants causing late-onset disorders for which reporting can be expected to cause an undeniable health benefit, such as those listed in the ACMG SF v3.0 list [14]. Fetal (and maternal) carriership for X-linked recessive disorders will be reported as well, as it can be of relevance for future pregnancies of both mother and child. On the other hand, variants causing late onset disease without actionability and carriership for autosomal recessive disorders will not be communicated. The turn-around-time (TAT) was nationally set at eight weeks for ongoing pregnancies.

## 3. Results

**Figure 1:**
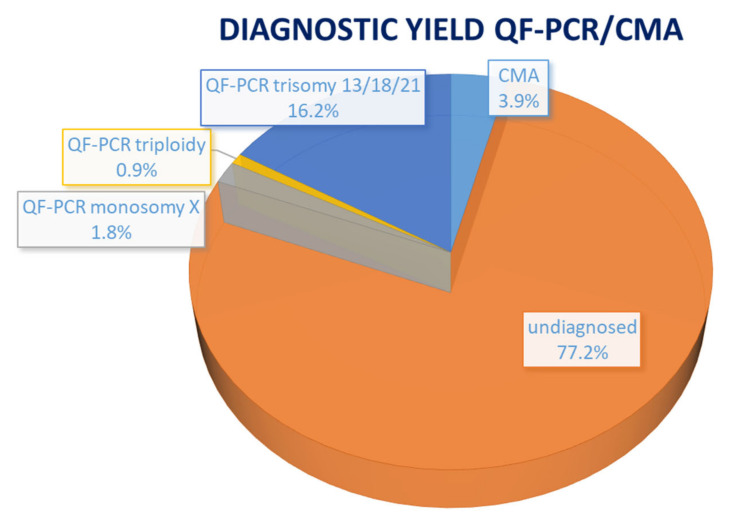
Diagnostic yield of QF-PCR/CMA in the prenatal context. QF-PCR: quantitative fluorescent polymerase chain reaction; CMA: chromosomal microarray.

The Center of Medical Genetics Antwerp, which is one of the eight genetic centers in Belgium, processes about 400 invasive prenatal samples on a yearly basis. In our center, the routine approach to determine the genetic etiology in case of fetal ultrasound anomalies, regardless of the gestational age, follows a sequential approach: first, we perform a quantitative fluorescent PCR (QF-PCR) for exclusion of the common aneuploidies (trisomy 13, 18, 21, sex chromosomal aneuploidies) and triploidy as well as for determination of maternal cell contamination and fetal identity through comparison to the maternal profile. Next, CNV detection by CMA is performed, more precisely a SNP (single nucleotide polymorphism) array with a 400 kb resolution. However, this combined approach yields a diagnosis in less than 25% of cases: on 3453 analyses that were performed over the past nine years, QF-PCR and SNP array were positive in 786 cases (22.8%), among which 557 with a trisomy (70.9% of positive and 16.1% of total cases), 30 with a triploidy (respectively 3.8% and 0.87% of cases), 62 with monosomy X (respectively 7.9% and 1.8% of cases) and 134 with a subchromosomal pathogenic anomaly (respectively 17% and 3.9% of cases) (Figure 1).

**Figure 2:**
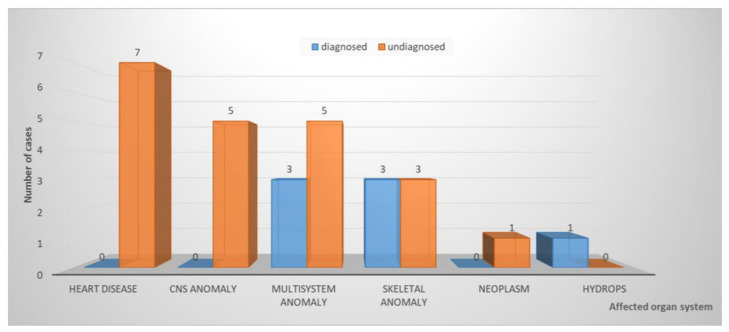
Distribution of fetal WES cases according to organ system. CNS: central nervous system.

**Figure 3:**
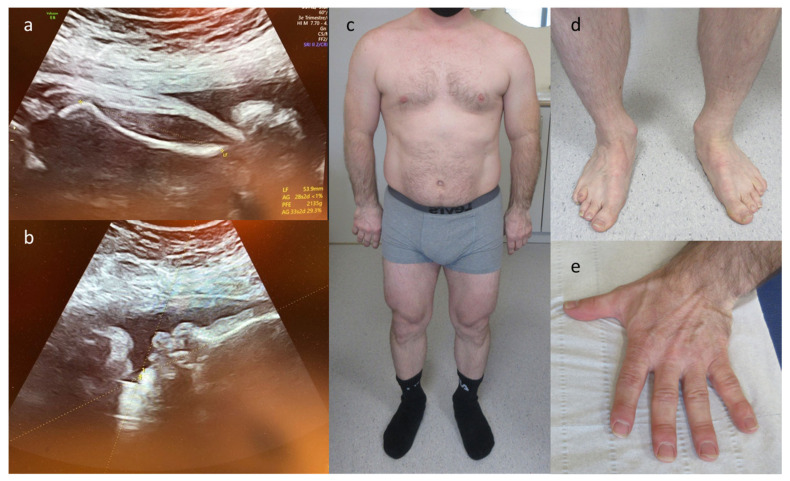
Phenotype of fetus and father with Stickler syndrome due to a *COL2A1* variant. (**a**) 3rd trimester ultrasound showing femoral shortening and bowing; (**b**) 3rd trimester ultrasound showing pathological lower facial angle at 44° corresponding to microretrognathia; (**c**) Affected father: short and stocky appearance; (**d**) Feet of affected father; (**d**) Feet of affected father: right 4th toe and left 4th and 5th toes are proximally implanted and the lower limbs show bowing at the ankles; (**e**) Left hand of affected father: short hand and brachydactyly.

Since January 2021, our center offers the subsequent option of whole exome sequencing (WES). In 2021, WES was performed in our center on 28 prenatal cases showing structural anomalies on ultrasound (see Figure 2 and Supplementary Table S1). Twenty WES analyses were performed on DNA extracted from uncultured amniotic cells, six from cultured amniotic cells and two from chorion villi. In all but two cases, an ‘open’ WES was performed, as the ultrasound anomalies did not allow the selection of a predefined gene panel; for the two remaining cases, the analysis was restricted to our skeletal dysplasia gene panel of 436 genes (see Supplementary Table S2 for the composition of the panel). All cases passed our quality score (capture of more than 95% of the exome with at least 20× coverage). Pathogenic or likely pathogenic variants were identified in seven out of 28 (25%) cases: two de novo variants, four autosomal recessive and one paternally inherited (from an affected parent) (see Table 1). In three fetuses with skeletal anomalies, WES detected respectively a dominantly inherited *COL2A1* variant (see Figure 3), a homozygous *FANCG* variant and a de novo *KMT2D* variant. The fetus with the *COL2A1* variant displayed rhizomelic shortening and bowing of the long bones, microretrognathia and clenched hands-on prenatal ultrasound. The fetus with the *FANCG* variant came to attention through intrauterine growth restriction (IUGR), thumb hypoplasia on the left hand and absent thumb on the right hand. The fetus with the *KMT2D* variant had talipes equinovarus as well as abnormal placing of the ears.

**Figure 4:**
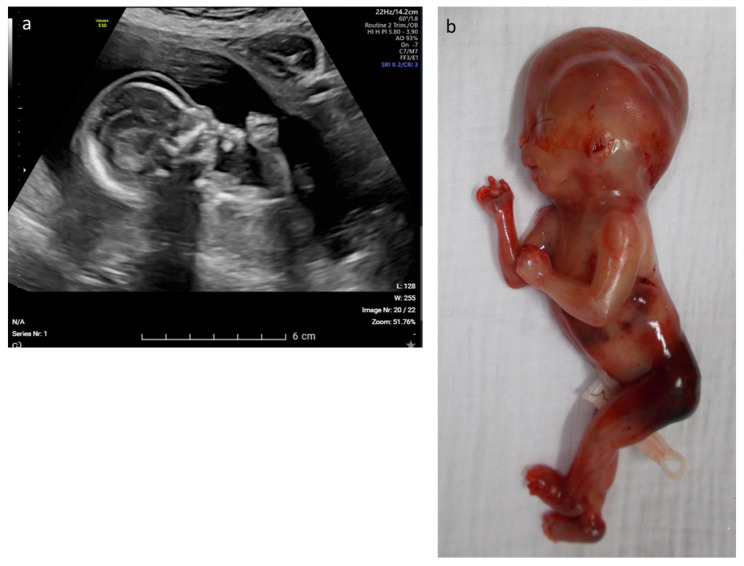
Fetus with a homozygous *MUSK* variant causing Fetal akinesia deformation sequence 1. (**a**) Ultrasound at 20 weeks, showing subcutaneous edema; (**b**) Clinical picture: rocker-bottom feet and hydrops fetalis.

Three fetuses with multisystem anomalies, defined as the presence of at least two major anomalies in different anatomical systems, carried respectively a homozygous *MUSK* variant (see Figure 4), a homozygous *CHRNA1* variant and compound heterozygous *THOC6* variants. The pregnancy with the *MUSK* variant was suspicious since it was the second pregnancy of this couple with fetal hydrops. The first was terminated and no genetic analyses had been performed; in the current, the evolution towards a more severe phenotype with fetal akineseia and abnormal position of the lower limbs justified exome sequencing. The fetus with the *CHRNA1* variant came to attention because of multiple congenital malformations, namely retrognathia, diffuse subcutaneous edema, increased nuchal fold, clenched fingers, bell-shaped thorax and bilateral rocker bottom feet. The fetus with the *THOC6* variants displayed Tetralogy of Fallot, cerebellar hypoplasia, mild ventriculomegaly and hypospadias. The last positive case was a de novo *RIT1* variant in a fetus with bilateral hydrothorax, ascites, generalized subcutaneous edema and polyhydramnion. All but one variant was classified as pathogenic; the missense variant in *CHRNA1* was classified as likely pathogenic as it had not been described in the literature, but was deemed pathogenic by prediction scores and fit the phenotype. Additionally, a pathogenic incidental finding in *STXBP1* was reported in one case that also carried a pathogenic variant which explained the phenotype (*RIT1*). In all but one case, the parents opted for a termination of the pregnancy (see Table 1). The exception was the fetus with the paternally inherited *COL2A1* variant. At birth, the baby showed—in addition to the prenatally observed anomalies—cleft palate, atrial septal defect, pathological auditory evoked potentials and ophtalmological abnormalities compatible with Stickler syndrome. At 4 months, her length is at P10 and some additional facial dysmorphisms, such as narrow palpebral fissures, long philtrum, thin upper lip and full cheeks, become apparent.

When grouped based on the organ system(s) involved (see also Mellis et al. 2022 [4]), the highest diagnostic yield was obtained in case of skeletal anomalies (three out of six cases or 50%) or multisystem anomalies (three out of eight cases or 37.5%). No diagnosis was found in seven cases with heart disease and five with a central nervous system anomaly (Figure 2).

## 4. Discussion

In Belgium, prenatal WES in a diagnostic setting is publicly funded and fully reimbursed. National guidelines describing the inclusion criteria, pre- and posttest counseling and the filtering and reporting strategy have been developed by a committee of laboratory and clinical geneticists and are publicly available on the website of the Belgian College for Human Genetics and Rare Diseases (www.college-genetics.be (accessed on 1 December 2022)). However, prenatal WES has not been implemented widely, as many hurdles still remain. Issues involve (1) the quality and quantity of the starting material; (2) the short TAT; (3) the interpretation of variants; (4) the ethical perspective.

In our center, no problems with DNA quality were encountered—for all samples, DNA was extracted in-house according to an accredited protocol that yields high quality DNA. For some of the samples, insufficient DNA was obtained upon extraction from uncultured amniocytes and a second DNA extraction from cultured amniocytes was required. As a precaution, we always culture part of the amniocytes. First, this provides a back-up source of DNA, although we need as little as 2 ng of starting material for QF-PCR, 20 ng for SNP array and 50 ng for WES. Second, during the culturing process, growth of amniocytes is enhanced, but that of peripheral blood cells is not, which is an advantage in case the QF-PCR on the DNA extracted from the uncultured amniocytes shows maternal cell contamination [15]. Although the TAT for prenatal WES has been set nationally at eight weeks, we lowered it to 4 weeks, allowing timely decision-making for the ongoing pregnancy; couples might either consider termination of pregnancy when a genetic diagnosis is established or the etiology could guide the obstetric and neonatal management. Both the library prep and the sequencing run are performed biweekly; analysis and interpretation take maximally 1 additional week, including multidisciplinary discussions on variant interpretation or reporting. All cases were analyzed as trio (index and both parents), which allows filtering of de novo, autosomal recessive and X linked variants. Additionally, we use a phenotype-driven software package for variant prioritization (Moon, Invitae) to detect inherited variants that fit the phenotype (e.g., in imprinted genes or with a mosaic or presumably unaffected carrier parent) and, in case of panel-based analysis, variants in genes outside the panel. Apart from trio analysis, other steps to limit the number of variants that require classification are minor allele frequency in the GnomAD database and our own database, location (only exonic variants and variants in the splice regions are considered) and allelic ratio of the mutant versus wild-type allele. Extensive phenotyping is key to interpretation of the remaining variants. In our center, we developed a database where clinicians can enter the phenotype as HPO (Human Phenotype Ontology) terms, allowing for structured phenotyping.

Correlating the genotype with the prenatal phenotype was challenging. In general, the fetal phenotype of many conditions has not been well described and may deviate quite substantially from the known postnatal phenotype. Cataloguing the phenotype of prenatal-onset syndromes is of utmost importance to guide healthcare providers in recognizing these syndromes at an early stage and know their evolution throughout the pregnancy [16,17]. The correlation was the most obvious in the fetus with the *RIT1* mutation (case 23), associated with Noonan type 8 (OMIM# 615355): hydrops, ascites and hydrothorax, which were all present in this fetus, are frequent ultrasound markers in RASopathies.

Given the genotypic heterogeneity of Noonan syndrome and by extension non-immune hydrops fetalis [18], WES or whole-genome sequencing are powerful diagnostic tools for these diseases [19]. This fetus also carried a de novo pathogenic variant in *STXBP1*, that was reported as incidental finding because of its association with developmental and epileptic encephalopathy 4 (OMIM# 612164). Given the severe phenotype of seizures, profoundly impaired psychomotor development, limited or absent ability to walk, spastic quadriplegia and poor or absent speech, prenatal testing in a future pregancy is warranted as parental gonadal mosaicism cannot be ruled out.

In case of a suspicion of a fetal skeletal dysplasia, the value of adding WES to the prenatal diagnostic tools has been demonstrated before [20,21,22]; this was confirmed in our cohort, with three out of six cases (50%) being solved by WES. The fetus with Stickler syndrome type I (OMIM# 108300) caused by a heterozygous *COL2A1* missense mutation (case 10) displayed rhizomelic shortening and bowing of the long bones as well as microretrognathia and clenched hands on ultrasound (Figure 3a,b). The mutation was paternally inherited, manifesting in the to that point undiagnosed father with severe myopia, hearing disorder, short stature, retrognathia, a nasal voice, tibia bowing, platyspondyly, coxofemoral dysplasia, hyperlordosis and rhizomelic shortening of the long bones (Figure 3c–e). Analysis of the paternal grandparents demonstrated that the mutation arose de novo in the father. The second fetus with skeletal anomalies (case 1) was diagnosed with Kabuki syndrome as a result of a de novo stop mutation in *KMT2D* (OMIM# 147920). Kabuki syndrome shows prenatal phenotypic heterogeneity, with ultrasound abnormalities that are non-specific. The most frequent ultrasound features include cardiac anomalies (49.4%), followed by polyhydramnios (28.9%), genitourinary anomalies (26.5%), single umbilical artery (15.7%), intrauterine growth restriction (14.5%) and hydrops fetalis/pleural effusion/ascites (12.0%) [23]. The fetus in our cohort showed only bilateral talipes equinovarus and abnormal ears, illustrating the broad fetal phenotypic heterogeneity. The third fetus (case 8) presented with intrauterine growth restriction, oligodactyly of the left hand and a hypoplastic ray of the right hand and was diagnosed with Fanconi anemia (FA) due to a homozygous *FANCG* nonsense variant (OMIM# 614082). FA is an autosomal recessive disorder with both phenotypic and genotypic heterogeneity, but major birth defects such as skeletal malformations (mainly uni- or bilateral radial ray anomalies), microcephaly, genitourinary malformations and intrauterine growth restriction are present in 75% of the cases. Consequently, these findings in the prenatal setting are suggestive of FA, although absence of skeletal anomalies does not exclude FA [24]. Radial ray defects, as present in this fetus, can be associated with various disorders, but in combination with IUGR or other MCA, it is indicative of FA [25,26]. Rapid WES in case of skeletal anomalies allows differentiating between isolated and syndromic forms, which is key to counseling the parents.

Of eight fetuses with multisystem aberrations in our series, three (37.5%) were positive. The first (case 26) was compound heterozygous for four known missense variants (of which three on the maternal allele that have been described as a pathogenic haplotype) in *THOC6*, causing Beaulieu-Boycott-Innes syndrome (OMIM# 613680). There are seven reports of prenatally diagnosed Beaulieu-Boycott-Innes syndrome with variable clinical findings, such as IUGR, cerebral malformations, genito-renal abnormalities, cystic hygroma, retrognathia and suspicion of ventricular septal defect [27]. Our case showed olivopontocerebellar hypoplasia, tetralogy of Fallot and hypospadias on ultrasound. The second fetus (case 28) was diagnosed with a homozygous missense variant in a receptor tyrosine kinase (*MUSK*). For fetal akinesia deformation sequence 1 (FADS1), caused by homozygous *MUSK* mutations (OMIM# 208150), prenatal diagnosis is based on multiple contractures, reduced motility, flattening of facial profile and—with increasing gestational age—IUGR, reduced cardiothoracic ratio and polyhydramnios [28]. The ultrasound features present in our case (hydrops, hydrothorax, ascites, fetal akinesia, hypotonia, rocker-bottom feet; Figure 4) fit the described prenatal phenotype. The third fetus (case 12) presented with nuchal translucency, edema, rocker-bottom feet, aberrant chest and ribs and retrognathia and was diagnosed with a homozygous *CHRNA1* missense variant. Recessive mutations in the *CHRNA1* gene result in lethal multiple pterygium syndrome (LMPS; OMIM# 253290). LMPS displays a heterogeneous range of prenatal manifestations that generally include cystic hygroma, pulmonary hypoplasia, cleft palate, cryptorchidism, joint contractures, fetal akinesia, heart defects, growth restriction and intestinal malrotation [29]. In retrospect, the phenotype of this fetus fits the LMPS syndrome, but a clinical diagnosis remains challenging in the prenatal stage.

In total, WES was able to pinpoint the cause of the fetal anomalies in 25% of cases (7 out of 28). Among the seven positive cases, two were de novo, four recessive and one paternally inherited (from an affected parent). Multidisciplinary genetic counseling of the prenatal results was performed and except for the parents with the fetus diagnosed with paternally inherited Stickler syndrome, all chose to terminate the pregnancy after approval by the ethical committee of the University Hospital Antwerp. For all cases, the decision to terminate was based on the WES result, effectively demonstrating its use in the prenatal setting. For five out of seven families (71.4%), the recurrence risk is high and preimplantation or prenatal invasive genetic testing can be offered in future pregnancies. For the family with the dominantly inherited variant, testing in first-degree relatives of the father can be considered as well. In the families with a de novo variant, genetic testing in future pregnancies should be discussed because of the possibility of parental gonadal mosaicism.

Ethically, the most demanding issue is the possibility of incidental findings in both fetus and parents. Although rigorous filtering can reduce the number of incidental findings, they can never be fully excluded as this would jeopardize the identification of the primary variant(s) explaining the phenotype. Therefore, a genetic pretest counseling as well as informed consent by both parents are mandatory, so that they are well aware of the possible outcomes. The only incidental finding we encountered was a de novo mutation in *STXBP1*, associated with developmental and epileptic encephalopathy type 4, in the fetus carrying the *RIT1* mutation. In case of future prenatal invasive testing, presence of both the *RIT1* and the *STXBP1* mutation can be evaluated.

The recent update of the ISPD position statement on prenatal WES states that although the available data is insufficient to recommend which categories of abnormalities warrant sequencing, there are ‘sufficient data to begin differentiating diagnostic yields by specific organ system or number of organ systems affected’ [9]. Our results confirm their findings that prenatal WES holds great promise for pregnancies with skeletal or multisystem anomalies. In our hands, prenatal WES was less successful in foetuses with cardiac and CNS abnormalities, but the number of cases in this study is too low to draw any definitive conclusions. It can be expected that, based on the contribution of this and other manuscripts describing the results of WES in the prenatal context, uniform guidelines on the indications for which to consider WES will follow in the near future.

## 5. Conclusions

Our data set, although limited, clearly shows the added value of WES in the prenatal setting in case of MCA. The diagnostic yield of 25% demonstrates that the rigorous selection of prenatal cases according to our national guidelines is effective; yield is highest in cases with skeletal or multisystem anomalies. Furthermore, our findings demonstrate that WES can be implemented in a medium-throughput diagnostic lab with little failures and an acceptable TAT, effectively expanding the diagnostic portfolio that can be offered to future parents.

## Figures and Tables

**Table 1:** Prenatal cases for which WES demonstrated a likely pathogenic or pathogenic variant. For each case, the fetal phenotype and the organ system involved are described and the affected gene and variant, the inheritance mode, the associated syndrome and the outcome of the pregnancy are listed. AD: autosomal dominant; AR: autosomal recessive; hom: homozygous; IF: incidental finding; IUGR: intrauterine growth restriction; LB: live birth; mat: maternal; NT: nuchal translucency; pat: paternal; path: pathogenic; TOP: termination of pregnancy.

Case No.	Phenotype	Phenotypic Group	Gene	Variant	Inheritance	Classification	Associated Syndrome	Outcome
1	Abnormal ears, bilateral talipes equinovarus	skeletal	*KMT2D*	c.450G > A p.(Trp150*)	AD–de novo	path	Kabuki syndrome 1 (OMIM# 147920)	TOP
8	IUGR, oligodactyly left hand, hypoplastic ray right hand	skeletal	*FANCG*	c.115C > T p.(Arg39*)	AR–hom	path	Fanconi anemia, complementation group G (OMIM# 614082)	TOP
10	Rhizomelic shortening and bowing of the long bones, microretrognathia and clenched hands	skeletal	*COL2A1*	c.2710C > T p.(Arg904Cys)	AD–pat	path	Stickler syndrome type I (OMIM# 108300)	LB
12	Edema, rocker bottom foot, retrognathia, abnormal thorax and ribs, increased NT	multisystem	*CHRNA1*	c.548A > G p.(Asp183Gly)	AR–hom	likely path	Multiple pterygium syndrome, lethal type (OMIM# 253290)	TOP
23	Hydrops, acites, hydrothorax	hydrops	*RIT1*	c.297T > A p.(Phe99Leu)	AD–de novo	path	Noonan syndroom 8 (OMIM# 615355)	TOP
			*STXBP1*	c.875G > A p.(Arg292His)	AD–de novo	path (IF)	Developmental and epileptic encephalopathy type 4 (OMIM# 612164)	
26	Olivopontocerebellar hypoplasia, tetralogy of Fallot, hypospadias	multisystem	*THOC6*	c.298T > A p.(Trp100Arg)	AR–het (mat)	path	Beaulieu-Boycott-Innes syndrome (OMIM# 613680)	TOP
			*THOC6*	c.700G > C p.(Val234Leu)	AR–het (mat)	path		
			*THOC6*	c.824G > A p.(Gly275Asp)	AR–het(mat)	path		
			*THOC6*	c.569G > A p.(Gly190Glu)	AR–het (pat)	path		
28	Fetal akinesia, hypotonia, rocker-bottom feet, hydrops, hydrothorax, ascites	multisystem	*MUSK*	c.2201G > T p.(Gly734Val)	AR–hom	likely path	Fetal akinesia deformation sequence 1 (OMIM# 208150)	TOP

## Data Availability

All data are archived in the patient files.

## Supplementary Materials

The following supporting information can be downloaded at https://www.mdpi.com/article/10.3390/diagnostics13050860/s1: Table S1: Fetal phenotype and genotype; Table S2: Composition of the skeletal dysplasia gene panel. Legend to Supplementary Table S1; This table contains the 28 fetal cases on whom WES was performed. For each case, the fetal phenotype and the organ system involved are described, as well as the sample type from which the DNA was extracted, the applied gene panel and the result. For the positive cases, the gestational age at ultrasound, the affected gene and variant, the inheritance mode, the associated syndrome, the fetal phenotype as described in the literature and the outcome of the pregnancy are listed as well. AC: amniocytes: AD: autosomal dominant; AR: autosomal recessive; CNS: central nervous system; CV: chorionic villi; hom: homozygous; IF: incidental finding; IUGR: intrauterine growth restriction; LB: live birth; mat: maternal; NT: nuchal translucency; pat: paternal; path: pathogenic; TOP: termination of pregnancy; US: ultrasound; VSD: ventricular septal defect; WES: whole exome sequencing; WESSD: WES with skeletal dysplasia panel; Legend to Supplementary Table S2. This table lists the genes included in the skeletal dysplasias gene panel, that was used for analysis of cases 6 and 10.
